# TLS and immune cell profiling: immunomodulatory effects of immunochemotherapy on tumor microenvironment in resectable stage III NSCLC

**DOI:** 10.3389/fimmu.2024.1499731

**Published:** 2024-12-11

**Authors:** Chaopin Yang, Jinqi You, Yizhi Wang, Si Chen, Yan Tang, Hao Chen, Haoran Zhong, Ruyue Song, Hao Long, Tong Xiang, Ze-Rui Zhao, Jianchuan Xia

**Affiliations:** ^1^ State Key Laboratory of Oncology in South China, Guangdong Provincial Clinical Research Center for Cancer, Collaborative Innovation Center for Cancer Medicine, Sun Yat-sen University Cancer Center, Guangzhou, Guangdong, China; ^2^ Department of Biotherapy, Sun Yat-Sen University Cancer Center, Guangzhou, Guangdong, China; ^3^ Department of Thoracic Surgery, Sun Yat-Sen University Cancer Center, Guangzhou, Guangdong, China

**Keywords:** NSCLC, immunochemotherapy, tertiary lymphoid structures (TLSs), the axis of PD-L1+CD11c+ cells and PD1+CD8+ T cells, CCR7+CD4+ T cells, CD38+CD8+ T cells

## Abstract

**Background:**

The use of programmed death-1 (PD-1) inhibitors in the neoadjuvant setting for patients with resectable stage III NSCLC has revolutionized this field in recent years. However, there is still 40%-60% of patients do not benefit from this approach. The complex interactions between immune cell subtypes and tertiary lymphoid structures (TLSs) within the tumor microenvironment (TME) may influence prognosis and the response to immunochemotherapy. This study aims to assess the relationship between immune cells subtypes and TLSs to better understand their impact on immunotherapy response.

**Methods:**

This study initially compared the tertiary lymphoid structures (TLSs) density among patients who underwent immunochemotherapy, chemotherapy and upfront surgery using 123 tumor samples from stage-matched patients. Multiplex immunohistochemistry (mIHC) was employed to analyze the spatial distribution of PD-L1+CD11c+ cells and PD1+CD8+ T cells within TLSs. Cytometry by time-of-flight (CyTOF) was used to assess immune cell dynamics in paired biopsy and resection specimens from six patients who underwent immunochemotherapy. Key immune cells were validated in newly collected samples using flow cytometry, mIHC, and *in vitro* CAR-T cells model.

**Results:**

Patients who underwent neoadjuvant chemotherapy or immunochemotherapy exhibited increased TLSs compared to those who opted for upfront surgery. The TLS area-to-tumor area ratio distinguished pCR+MPR and NR patients in the immunochemotherapy group. Spatial analysis revealed variations in the distance between PD-L1+CD11c+ cells and PD1+CD8+ T cells within TLSs in the immunochemotherapy group. CyTOF analysis revealed an increase in the frequency of key immune cells (CCR7+CD127+CD69+CD4+ and CD38+CD8+ cells) following combined therapy. Treatment responders exhibited an increase in CCR7+CD4+ T cells, whereas CD38+CD8+ T cells were associated with compromised treatment effectiveness.

**Conclusions:**

Immunochemotherapy and chemotherapy increase TLSs and granzyme B+ CD8+ T cells in tumors. The TLS area-to-tumor ratio distinguishes responders from non-responders, with PD-L1+ dendritic cells near CD8+PD-1+ T cells linked to efficacy, suggesting that PD-1 inhibitors disrupt harmful interactions. Post-immunochemotherapy, CD8+ T cells increase, but CD38+CD8+ T cells show reduced functionality. These findings highlight the complex immune dynamics and their implications for NSCLC treatment.

## Background

More than 50% of patients with resectable, locally advanced non-small cell lung cancer (NSCLC) experience disease relapse within two years following surgery (1, 2). Efforts have been made to eliminate micrometastases, which can lead to recurrence, by employing perioperative therapy during surgery. However, prior trials investigating neoadjuvant chemotherapy or chemoradiation following surgical resection have yielded limited success, with median pathologic complete response (pCR) rates as low as 4% (range: 0 to 16%) and only a 4-5% increase in the overall survival rates of patients with stage III NSCLC (3–5), leading to a 5-year overall survival rate of 25 to 38% (3). The use of programmed death-1 (PD-1) inhibitors in the neoadjuvant setting has revolutionized this field in recent years. In the phase 2 NADIM trial, neoadjuvant nivolumab plus chemotherapy achieved an unprecedently high pCR rate of 57% in stage III NSCLCs (4). This result was further confirmed in the phase 2 randomized NADIM-II study, where immunochemotherapy significantly improved the pCR rate compared with chemotherapy (37% *vs*. 7%) as a neoadjuvant treatment (5). Similarly, in the phase III randomized CheckMate-816 trial, neoadjuvant nivolumab plus chemotherapy resulted in a significantly greater percentage of patients with resectable NSCLCs achieving pCR than chemotherapy alone (24.0% *vs*. 2.2%) (6). Our own previous phase II trial also demonstrated the efficacy of neoadjuvant PD-1 and chemotherapy in Asian patients with stage III NSCLCs, yielding a pCR rate of 50% (7). Similar results were also observed in a study by another group (8).

Among these exciting developments, there is growing interest in understanding the intricate crosstalk of immune cells within the tumor microenvironment (TME). Emerging evidence underscores the pivotal role of immune cells in the TME (9, 10); however, resistance to hyperprogressive disease in patients treated with checkpoints has also been reported (11). Given its promising clinical activity and the presence of some paradoxical treatment effects, the mechanism of neoadjuvant immunotherapy in NSCLCs needs further clarification. Understanding these mechanisms will improve our approach to treating locally advanced NSCLC (12–14). Several studies have characterized the lung TME at single-cell resolution in both early- and late-stage NSCLC, providing a comprehensive characterization of the cell types within the TME at high resolution (14–17). As far as we know, very few studies have explored single-cell analysis before and after neoadjuvant treatment, especially under the context of neoadjuvant immunochemotherapy.

Tertiary lymphoid structures (TLSs) are ectopic lymphoid organs that develop in chronic inflammatory autoimmune infectious diseases, transplanted organs, inflammatory disorders and tumors (18). The morphology of TLSs is similar to that of secondary lymphoid organogenesis, such as lymph nodes (19), which are the classical site of the generation of efficient adaptive immune response against cancer. Thus, TLSs may exhibit antitumor effects. The clinical benefits of TLSs and several key immune cells have been described (20–22). An increase in TLS size has opened up a new field of immune-oncology treatment, with reports suggesting treatment benefit (23–27). However, the functional mechanisms of TLSs remain unclear.

In this study, we investigated the impact of neoadjuvant immunochemotherapy and chemotherapy on TLSs in patients with resectable stage III NSCLC. Additionally, we used cytometry by time-of-flight (CyTOF) to identify key immune cell populations and analyzed primary tumors (PTs) in patients with NSCLC who were treated with a preoperative PD-1 inhibitor along with chemotherapy. Our study further identified several key immune cell subtypes in this context using multiple immunofluorescence techniques and flow cytometry, and validated the findings in newly collected samples. This research highlights potential biomarkers and mechanisms underlying treatment response in patients with resectable stage III NSCLC who receive neoadjuvant immunochemotherapy.

## Materials and methods

### Patients and human tumor samples

All patients enrolled in this study had resectable stage III (stage IIIA or T3-4N2 IIIB) NSCLCs according to the 8^th^ edition of TNM staging system (28). Patients received neoadjuvant treatment with intravenous toripalimab (240 mg) on day 1, carboplatin (area under curve 5) on day 1, and pemetrexed (500 mg/m^2^ for adenocarcinoma) or nab-paclitaxel (260 mg/m^2^ for other histologic types) on day 1 of each 21-day cycle for three cycles, as described in our previous clinical trial conducted between 2019 and 2021.

Seventeen patients were included in this study for CyTOF analysis, and six patients with paired tissue samples were further analyzed. Two patients had only post-treatment tissues available because their immune cells failed to meet the minimal requirement for CyTOF analyses in baseline biopsies. Nine patients were excluded due to tumor progression, treatment regimens other than immunochemotherapy, or failure to meet the minimum standard for CyTOF analysis. Ultimately, six patients had primary tumors (PTs), and their paired lymph nodes (LNs) were collected before and after neoadjuvant therapy (**Table 1**) for CyTOF analysis. Among them, one was a responder (major pathological response [MPR] or pathological complete response [pCR]), and five were non-responders.

**Table 1:** Clinical characteristics of 17 NSCLC patients.

Enrollment or not	Neoadjuvant Treatment	Patient ID	EGFR/ALK	Age	Stage (TNM)	TILs (%)	Tumor		Time to progression (months)
						Pretreatment (Biopsied lesion)	Posttreatment		
N (number of TILs was not sufficient for CyTOF)	Anti-PD-1+Chemo	P001	0			Y(EBUS, butCD45+<2000)	N		
N (number of pretreatment TILs was sufficient, but the patient died due to coronary artery disease during treatment)	Anti-PD-1+Chemo	P002	0			Y(EBUS, CD45+>10000)	N		
N (number of TILs was not sufficient for CyTOF)	Anti-PD-1+Chemo	P003	0			NA	NA		
Y	Anti-PD-1+Chemo	P004	0	66	T2N2M0	Y (insufficient number of TILs)	Y, Surgery	pCR	NA
Y	Anti-PD-1+Chemo	P005	0	52	T2N2M0	Y	Y, Surgery	PR (PT RVT 45%, LN RVT 0%)	6.7
Y	Anti-PD-1+Chemo	P006	EGFR	63	T2N2M0	Y	Y, Surgery	PR (PT RVT 50%, LN RVT 70%)	NA
Y	Anti-PD-1+Chemo	P007	0	61	T2N2M0	Y	Y, Surgery	PR (PT RVT 90%, LN RVT 50%)	17.1
N	Tyrosine kinase inhibitor	P008	EGFR			Y (insufficient number of TILs)	Y, Surgery		
Y	Anti-PD-1+Chemo	P009	0	71	T3N2M0	Y	Y, Surgery	pCR	NA
Y	Anti-PD-1+Chemo	P010	EGFR	51	T2N2M0	Y	Y, Surgery	PR (PT RVT 80%, LN RVT 40%)	12.2
Y	Anti-PD-1+Chemo	P011	0	63	T3N2M0	Y	Y, Surgery	PR (PT RVT 30%, LN RVT 0%)	NA
Y	Anti-PD-1+Chemo	P012	0	59	T2N2M0	Y (insufficient number of TILs)	Y, Surgery	PR (PT RVT 30%, LN RVT 0%)	NA
N	NA, due to tumor progression	P013	0			Y	Y, Surgery		
N	NA, biopsy confirmed inflammatous	P014	0			Y	N		
N	Tyrosine kinase inhibitor	P015	ALK			Y	Y, Surgery		
N (number of TILs was not sufficient for CyTOF)	Tyrosine kinase inhibitor	P016	ALK			Y (insufficient number of TILs)	Y, Surgery		
N	NA, stage IV disease	P017	0			NA	N		

'NSCLC, non-small-cell lung cancer; N, No; NA, Not available; Y, Yes; EBUS, endobronchial ultrasonography. ^#^ PR, partial response; pCR, pathological complete response; SD, stable disease; PD, progressive disease RVT, residual viable tumor; PT, primary tumor; LN, lymph node.

The pathological response was categorized based on the percentage of viable tumor cells: pCR (0% viable tumor cells), MPR (> 0 and ≤ 10% viable tumor cells), and partial or no response (NR, >10% viable tumor cells). Nodal downstaging was defined as cN2 to ypN0/1. The treatment effect was evaluated by at least two experienced pathologists according to the respective consensus (29). To validate the findings, patients with similar stage who were treated with neoadjuvant immunochemotherapy (n=77), neoadjuvant chemotherapy (n=25), and upfront surgery (n=21) during the same period were consecutively enrolled.

### TLS evaluation

A total of 123 tumor samples, corresponding to the clinical tumor slides analyzed for TLS evaluation, were collected from patients treated with either immunochemotherapy, chemotherapy, or surgery. TLSs were evaluated according to a previous study (22). Briefly, whole-slide scanning images were obtained using a Polaris System (PerkinElmer, Waltham, Massachusetts, USA). TLSs were then quantified and analyzed using HALO image analysis software (IndicaLabs) and Rscript (Version 3.1) based on both H&E and CD20 immunohistochemical staining. In this study, the criteria used for the quantification of TLSs included (1) the total number of structures identified either within the tumor area or in direct contact with the tumor cells at the tumor margin (expressed as the numbers of TLSs per mm^2^ area) and (2) the total tumor area occupied by the TLSs, calculated as the ratio of the area of TLSs to the total tumor area.

To better understand the mechanisms through which TLSs contribute to NSCLC pathogenesis, we utilized TCGA data and TLS-related gene sets associated with T cells (CD3E, CD4, CD8A), B cell (CD19), and other immune markers, including PTPRC, CCL19, CCR7, CD28, LTA, CXCR3, IFNG, IL2, IL12B, IL15, TBX21, TNF, FASLG, GZMB, PRF1, CCL5, CCR2, CCR4, CCR5, CD40LG, CTLA4 and CSF2, based on a previous study (30). We then employed the ssGSEA algorithm using R packages (GSVA, GSEABase, and limma) to comprehensively evaluate the immunological characteristics of each sample in TCGA. Based on the median enrichment score, the samples were divided into high and low TLS groups.

### Sample collection and processing

Peripheral blood mononuclear cells (PBMCs) were isolated using Cell Separation Media solution (MD Pacific, Tianjin, China) as previously described. For tumor biopsy samples, CT-guided core needle biopsy and mediastinoscopy were used to obtain primary tumor (PT) samples at baseline. After tumor resection, a small block (approximately the size of a soybean) was collected and immediately stored at 4°C in tissue storage solution (Miltenyi, #130-100-008). Then, the samples were subsequently analyzed using CyTOF by PLTTECH (Zhejiang, China).

### CyTOF data acquisition and analysis

Briefly, the tumor samples were dissociated into single cells. Red blood cell lysis buffer was used to remove red blood cells, and dead cells were excluded. The antibodies used were pre-conjugated (Fluidigm, DVS Sciences) or purchased from Biolegend, Abcam, Thermo Fisher and R&D, and used according to the manufacturer’s instructions. In total, 42 metal-conjugated antibodies were used (**Table 2**). The protocol provided by Fluidigm (South San Francisco, California, USA) was followed. Living cells were selected by staining with cisplatin (Fluidigm, Kentucky, USA) and diluted to a concentration of 5 ×10^-3^ M. All metal-conjugated antibodies were titrated and mixed with the cell suspension in fluorescence-activated cell sorting (FACS) buffer (1xPBS+0.5% BSA) for 15 min. The cells were incubated with antibodies for 1 hour. The cells were rinsed and then collected using a Helio3 CyTOF mass cytometer (Fluidigm) at PLTTECH (Hangzhou, China) to detect the signal. CyTOF analyses were performed by PLTTECH, Inc. (Hangzhou, China) according to a previously described protocol (31). All classification and function markers were applied for clustering and visualization. Cells were annotated with classic markers. Subtype cells were defined by specific markers. The t-distributed stochastic neighbor embedding (t-SNE) algorithm was then applied to visualize the high-dimensional data in two dimensions. A heatmap of the normalized marker expression levels was generated. The ggplo2 R package was used to display the data.

**Table 2:** Mass cytometry antibody reagents.

Number	Protein	Clone	Lot Num.	Company	Metal isotope	surface or intracellular stain
1	CD45	HI30	304002	BioLegend	89Y	S
2	CD3	UCHT1	BE0231	Bio Cell	115In	S
3	CD68	Y1/82A	333802	BioLegend	139La	S
4	CD56	NCAM16.2	559043	BD	141Pr	S
5	TCRgd	5A6.E9	PLTTECH	PLTTECH	142Nd	S
6	CD196_CCR6	G034E3	353402	BioLegend	143Nd	S
7	CD14	M5E2	301810	BioLegend	144Nd	S
8	CD62L	DREG-56	304812	BioLegend	145Nd	S
9	CD123_IL_3R	6H6	306002	BioLegend	146Nd	S
10	CD15_SSEA_1	W6D3	323002	BioLegend	147Sm	S
11	CD19	HIB19	302214	BioLegend	148Nd	S
12	CD25_IL_2Ra	24212	MAB1020	RD	149Sm	S
13	CD274_PD_L1	29E.2A3	329702	BioLegend	150Nd	S
14	CD38	HIT2	303502	BioLegend	151Eu	S
15	CD27	O323	302802	BioLegend	152Sm	S
16	CD194_CCR4	L291H4	359402	BioLegend	153Eu	S
17	CD163	GHI/61	333602	BioLegend	154Sm	S
18	CD45RA	HI100	304102	BioLegend	155Gd	S
19	CD86	Fun-1	555655	BD	156Gd	S
20	CD183_CXCR3	G025H7	353750	BioLegend	157Gd	S
21	CD197_CCR7	G043H7	353222	BioLegend	158Gd	S
22	CD11c	BU15	337202	BioLegend	159Tb	S
23	CD33	WM53	303419	BioLegend	160Gd	S
24	CD152_CTLA_4	BN13	BE0190	Bio Cell	161Dy	S
25	CD69	FN50	310902	BioLegend	162Dy	S
26	CD138	DL101	352302	BioLegend	163Dy	S
27	CD185_CXCR5	RF8B2	552032	BD	164Dy	S
28	CD66b	G10F5	305102	BioLegend	165Ho	S
29	Perforin	B-D48	ab47225	Abcam	166Er	I
30	CD20	2H7	302302	BioLegend	167Er	S
31	CD24	ML5	311102	BioLegend	168Er	S
32	Ki67	SolA15	14-5698-82	eB	169Tm	I
33	CD127_IL_7Ra	A019D5	351302	BioLegend	170Er	S
34	IgD	IA6-2	348202	BioLegend	171Yb	S
35	CD273_PD_L2	24F.10C12	329610	BioLegend	172Yb	S
36	Granzyme B	QA16A02	372202	BioLegend	173Yb	I
37	CD279_PD_1	EH12.2H7	329926	BioLegend	174Yb	S
38	CD16	3G8	302014	BioLegend	175Lu	S
39	HLA-DR	L243	307612	BioLegend	176Yb	S
40	CD4	RPA-T4	300516	BioLegend	197Au	S
41	CD8a	RRA-T8	301018	BioLegend	198Pt	S
42	CD11b	M1/70	101202	BioLegend	209Bi	S

### Flow cytometry sorting

Single cells were collected from PTs as described previously. Briefly, PT tissues were cut into approximately 1-mm^3^ pieces in X-Vivo medium (Lonza, USA) and enzymatically digested with a MACS Tumor Dissociation Kit (Miltenyi Biotec) for 1 hour on a rotor at 37°C according to the manufacturer’s instructions. The dissociated cells were subsequently passed through a 40-μm cell strainer (BD) and centrifuged at 400 × g for 5 min. After the supernatant was removed, the pelleted cells were suspended in red blood cell lysis buffer (Beyotime, #C3702) at room temperature for 10 min to lyse the red blood cells. After washing with 1× PBS (Invitrogen), the cells were stained as described in the panels below. The purity of the sample was determined using flow cytometry on a Beckman MoFlo Astrios (Beckman, USA).

PBMCs were isolated using buffy coat solution (MD Pacific, Tianjin, China) as described previously. After erythrocyte lysis (Beyotime, #C3702, China), mononuclear cells were stained with the different panels below.

Two panels were used, as shown below:

Panel 1: Fixable viability stain 780 (FVS780, live/dead, BD, #565388), CD45-BUV395 (BD, #563792), CD3-AF700 (BD, #557943), CD4-FITC (BD, #555346), APC-CD69 (BD, #560711), CCR7-PE-Cy7 (BD, #557648), and CD127-BB700 (BD, #566398).

Panel 2: FVS-780 (live/dead), CD45-BUV395, CD3-AF700, CD8-FITC (BD, #555366), CD38-PE (BD, #555460), and PD-1-BV421 (BD, #562516).

### Intracellular cytokine staining assays

Both CD4+ and CD8+ sorted cells were stimulated using a leukocyte activation cocktail with BD GolgiPlug (BD, USA, #550583) and monensin (Biolegend, USA, #420701) for 5 hours. FVS780 was used to distinguish dead/live cells. For CD4+ T cells, intracellular cytokine staining for IFNγ-BV605 (BD, #562974), IL-4-PE-CF594 (BD, #565161) and IL-17 (BD, #563745) was applied to distinguish different Th1/Th2/Th17 cell types. For CD8-sorted cells, intracellular cytokines were stained with IFNγ-APC (BD, #562017), Granzyme B-BV421 (BD, #562641), and TNFα-PE (BD, #559321) to assess CD8+ T-cell function.

To determine apoptosis, CD133-specific CAR-T cells and CD38-overexpressing CAR-T cells were stained with annexin V and propidium iodide (PI) according to the manufacturer’s protocol (BD Biosciences) for flow cytometry analysis.

### Immunohistochemistry

The mounting slides were incubated at 65°C for 2.5 h. Samples were then subject to hydration; fixation in antigen retrieval solution (pH adjusted); treatment with 3% H_2_O_2_; blocking with goat serum; incubation with primary antibodies against granzyme B (CST, #46890, 1:200), CD20 (Abcam, #ab78237, 1:50), CD8 (OriGene #ZA-0508, working solution), and PD1 (CST, #86163), separately; and then incubation with secondary antibodies (Dako, Agilent). The slides were scanned using a digital pathology section scanner (KFBIO, Ningbo, China) to obtain a whole scan. The amplified view was photographed using microscopy (Nikon Eclipse Ni-U).

### Multicolor immunohistochemistry

The immunofluorescence (IF) markers used were grouped into three panels:

Panel 1 consisted of CD4 (Abcam # ab 133616, 1:500), CD69 (Abcam # ab 233395, 1:500), CCR7 (Abcam # ab 191575, 1:500), and CD127 (Abcam # ab 259806, 1:500) antibodies.

Panel 2 consisted of CD38 (Abcam # ab108403, 1:100), CD8 (OriGene#ZA-0508, working solution), and PD1 (Abcam # ab137132, 1:500) antibodies.

Panel 3 consisted of PD1 (Abcam, # ab52587, 1:100), CD8 (OriGene #ZA-0508, working solution), PDL1 (Abcam, # ab 205921, 1:100), and CD11c (Abcam, # ab 52632, 1:1000).

To confirm different cell types in FFPE specimens, a PANO 4-Colour IHC Kit (Cat# 10001100050, PANOVUE) or PANO 5-Colour IHC Kit (Cat# 10002100100) was used to perform multiplex immunofluorescence staining according to the manufacturer’s protocol. For details, antigen retrieval was performed with citric acid or EDTA solution in a microwave oven for 15 minutes. Then, the tissues were preincubated in blocking buffer at room temperature for 10 minutes. Specimens were incubated with different primary antibodies sequentially, followed by incubation with secondary antibody and signal amplification working solution at room temperature for 10 minutes. After all the markers were labelled, and DAPI (Sigma) was used to stain the cell nucleus. The whole-slide scanning fluorescence images were obtained using a Polaris System (PerkinElmer, Waltham, Massachusetts, US) and then analyzed using HALO image analysis software (IndicaLabs) Rscript (Version 3.1).

### Spatial proximity analysis

The digital images of multiplex fluorescence immunohistochemical staining were imported into Halo software v2.0 (Indica labs), and the stained cells were first subjected to DAB staining and quantified using the HALO multiplex algorithm v.1.2 to identify cell surface markers. The spatial analysis module of HALO, with a boundary of 20 μm, was used to specifically analyze the proximity of CD11c+PD-L1+ coexpressing cells and CD8+PD-1+ cells. When analyzing TLSs, the TLSs in the tumor area (intratumoral) and the adjacent area (peritumoral) of the tumor (within 30 μm adjacent) were selected, and five randomly selected TLSs were counted on the same slide.

### CAR-T-cell production

To generate CD133-specific CAR-T cells, we engineered a fusion protein encoding a fully human scFv derived from HW350341.1 in a previous study (32) linked to the human GMCSFRR leader peptide and used the CD28 sequence to replace the 41BB sequence as previously described (33).

### Cytotoxicity assays

The cytotoxicity of CAR-T cells transduced with a GFP or CD38 virus was determined using DELFIA Cell Cytotoxicity assays (PerkinElmer) according to the manufacturer’s protocol.

### Statistical analyses

All the statistical analyses were performed using GraphPad Prism (GraphPad Software Inc., San Diego, CA) except for the survival curve analysis. Mann−Whitney and Kruskal−Wallis tests were used to compare the density of infiltrating immune cells between different groups of patients. Survival analysis was performed using the Kaplan−Meier method and the log-rank test. Univariate and multivariate analyses were conducted using the Cox proportional hazards model to analyze prognostic factors. Survival analysis was performed using the survival R package, and p< 0.05 was considered to indicate statistical significance. All the statistical tests were two-sided, and the following differences were considered statistically significant: *p< 0.05, **p< 0.01, ***p< 0.001, and *p*< 0.0001.

## Results

### Immunochemotherapy or chemotherapy induces emergence of TLSs in tumors, with an increased number of granzyme B+ and CD8+ T cells in proximity to TLSs in the immunochemotherapy group, compared to the surgery-only group

**Figure 1:**
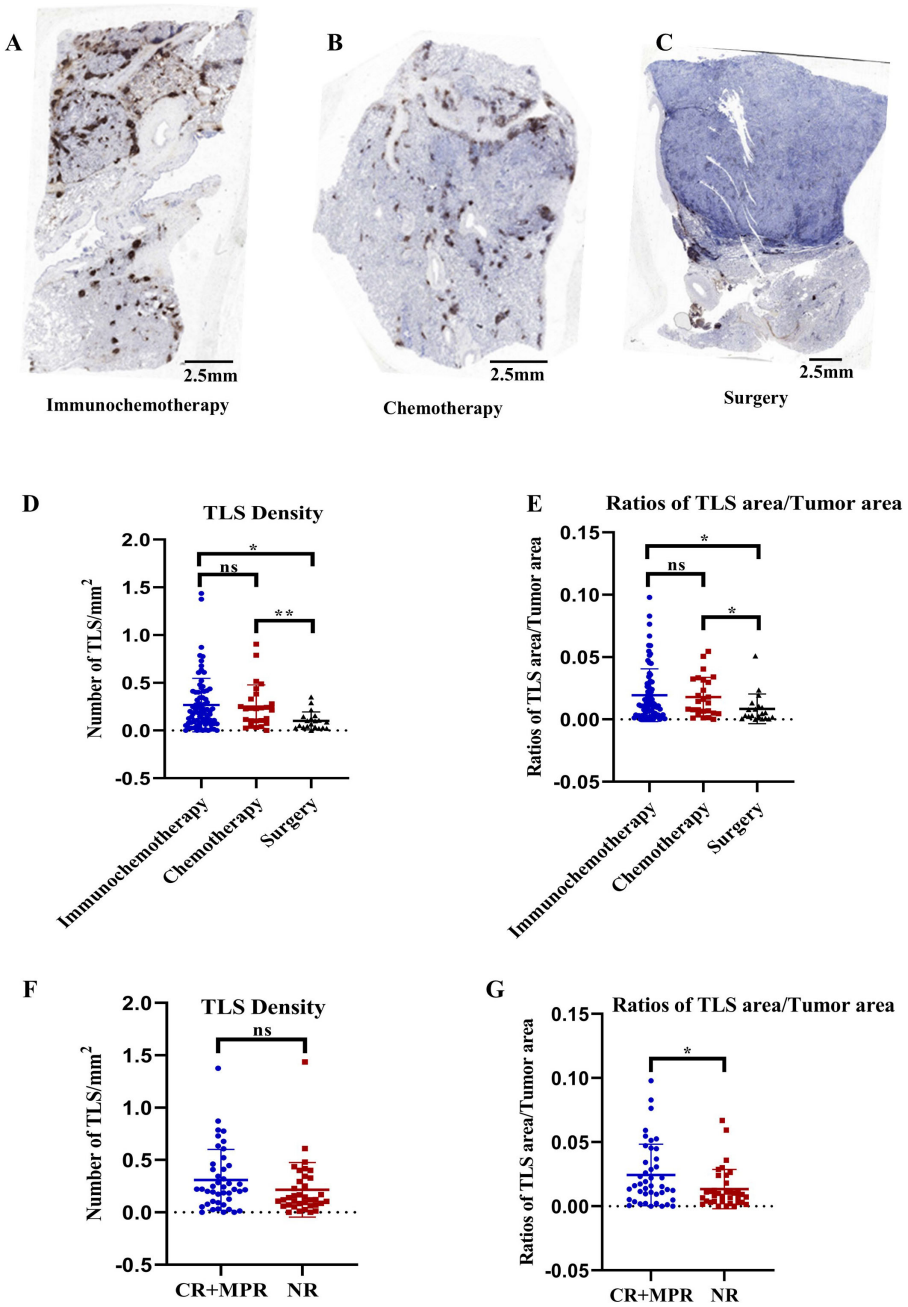
Correlations between pathological response and TLS characteristics. **(A–C)** Representative immunohistochemistry (IHC) images of tumor sections stained with CD20 from patients who received immunochemotherapy, chemotherapy, or upfront surgery, respectively. **(D, E)** Comparison of TLS density and TLS/tumor area ratios among the immunochemotherapy, chemotherapy, and upfront surgery groups. n≥21. **(F, G)** Comparison of TLS density and ratios of TLS/tumor area between responders (major pathological response [MPR] or pathological complete response [pCR]) and nonresponders in the immunochemotherapy group. The data are shown as the means ± SDs; n ≥25. Statistical significance was determined using one-way ANOVA with multiple comparisons in **(D, E)**. Mann−Whitney tests were performed to determine statistical significance **(F, G)**. *p < 0.05, **p < 0.01, ***p < 0.001, ns, not significant.

We stained 123 newly collected tumor samples from stage-matched patients (77 patients who received immunochemotherapy, 25 patients who received chemotherapy, and 21 patients who underwent upfront surgery) with a CD20 antibody to identify TLSs. The prognostic value and clinical relevance of TLSs were investigated. The number and area of TLSs in the immunochemotherapy and chemotherapy groups were significantly greater than those in the upfront surgery group (**Figures 1A-E**) (*p*=0.0103 and *p*=0.0076 in **Figure 1D**, *p*=0.0290 and *p*=0.0314 in **Figure 1E**, respectively), indicating that both immunochemotherapy and chemotherapy induced TLS formation. Furthermore, we found that the TLS area/tumor area ratio (*p*=0.02), but not the density (TLS number per unit area) (*p*=0.0788), could distinguish pCR+MPR from NR patients in the immunochemotherapy group (**Figures 1F, G**). We employed the ssGSEA algorithm using R packages (GSVA, GSEABase, and limma) to comprehensively evaluate the immunological characteristics of each sample included in the study based on TLS immune gene sets, as previously reported (30). Consistent with our results, the TLS score distinguished different prognostic groups of NSCLC patients (**Supplementary Figure S1**).

**Figure 2:**
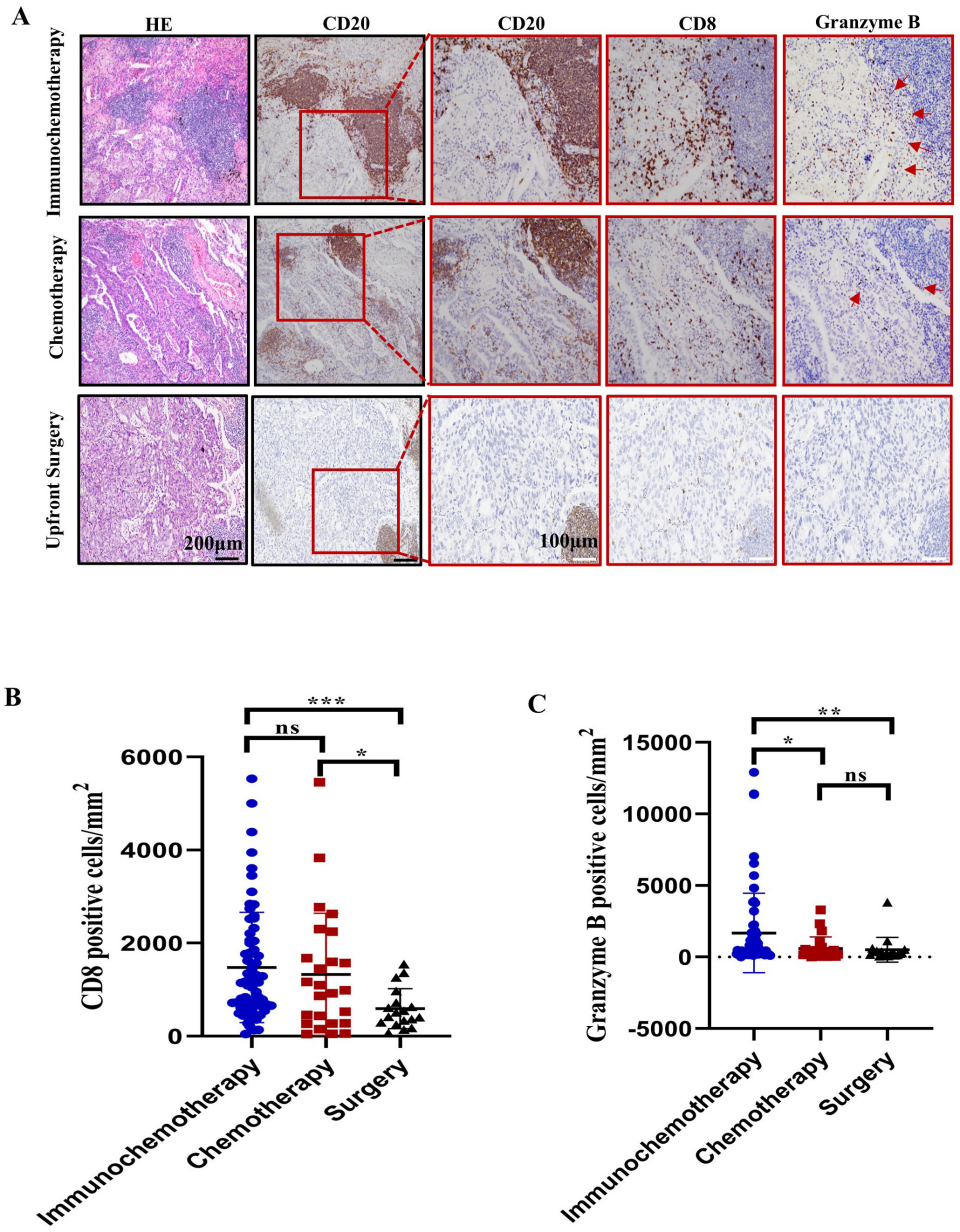
Characterization of CD8+ cells near tertiary lymphoid structures (TLSs) in the immunochemotherapy, chemotherapy and upfront surgery groups. **(A)** Representative images of HE, CD20, CD8, and granzyme B staining in the immunochemotherapy, chemotherapy, and upfront surgery groups at various magnifications. Black scale bars=200 μm, white scale bars=100 μm. **(B)** Bar plot showing the quantification of CD8+ lymphocytes in tumors corresponding to CD8+ T cells per square millimetre. **(C)** Bar plot showing the quantification of granzyme B in tumors per square millimetre. The data are shown as the means ± SDs; n ≥21. Statistical significance was determined using one-way ANOVA with multiple comparisons in **(B, C)**. *p < 0.05, **p < 0.01, ***p < 0.001, ns, not significant.

Furthermore, we performed immunohistochemistry to stain for CD8+ and intratumoral granzyme B. The results showed that CD8+ T-cell density was greater in the immunochemotherapy and chemotherapy groups than in the upfront surgery group (*p*=0.0002 and *p*=0.0282, respectively) (**Figures 2A, B**). An increased amount of granzyme B staining was noted in the immunochemotherapy group compared with the chemotherapy and surgery groups (*p*=0.03 and *p*=0.01, respectively) (**Figures 2A, C**). These results indicated that the presence of TLSs is associated with a higher number of functional CD8+ T cells compared to chemotherapy alone. Additionally, the PD-1 inhibitor in the immunochemotherapy group enhanced the adjuvant effect by increasing CD8+ T-cell cytotoxicity.

### PD-L1 expression is observed in CD68+ and CD11+ cells within TLSs, and the spatial colocalization of CD8+PD1+ with CD11c+PDL1+ cells in TLSs is clinically relevant to the treatment response in NSCLC patients

**Figure 3:**
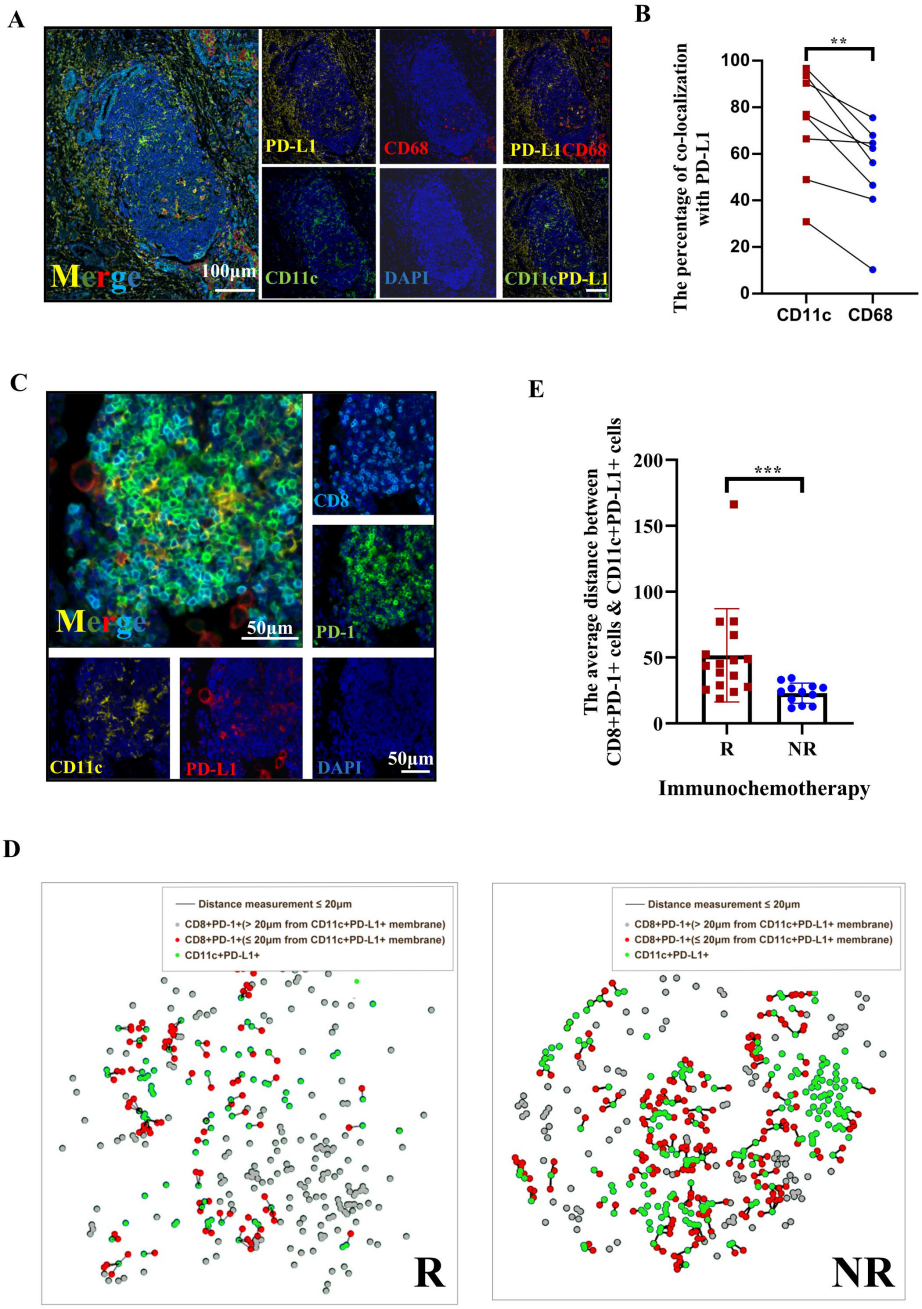
The spatial distribution of *PD1 CD8+ DC PDL1 colocalization* in tertiary lymphoid structures (TLSs) is an indicator of the response of patients with NSCLC following neoadjuvant immunochemotherapy. **(A)** Multiplex immunohistochemistry (mIHC) staining of a representative tumor section showing the coexpression of PD-L1 (yellow), CD68 (red), and CD11c (green) in TLSs, with nuclei counterstained with DAPI (blue). White scale bars=100 μm. **(B)** The percentages of CD11c+ cells and CD68+ cells among PD-L1+ cells. n=8. **(C)** Representative image of IHC and immunofluorescence staining of serial tissue sections after immunochemotherapy. The red arrow in the IHC image indicates the TLSs shown in the immunofluorescence images. The arrow indicates the area with high magnification/TLS. Digital markup image showing the color coding of CD8+ (cyan), PD-1 (green), PD-L1 (red), and CD11c (yellow). The peritumoral (PT) TLSs and intratumoral (IT) TLSs were annotated. Black and white scale bars=500 μm (left) and 50 μm (right), respectively. **(D)** Representative composite image depicting proximity analysis between CD8+PD-1+ and CD11c+PD-L1+ cells using the HALO software spatial analysis module in responders and nonresponders after treatment with immunochemotherapy. **(E)** Immunofluorescence analysis of the distance between CD8+PD-1/CD11c+PD-L1 interactions in PTs. n≥12. Paired t tests were performed to determine statistical significance, as shown in **(B)**. Mann−Whitney tests were performed, and the results are shown in **(E)**. *p < 0.05, **p < 0.01, ***p < 0.001, ****p < 0.0001, ns, not significant.

A previous study showed that PD-L1 expression on human tumor-infiltrating myeloid cells plays an indispensable role in the regulation of the T-cell response (34). The TLS/tumor area ratio indicates a positive response to immunochemotherapy in this study. Therefore, we examined how PD-1 inhibitors influence the PD-1–PD-L1 axis in TLSs. The colocalization of CD11c with PD-L1 and of CD68 with PD-L1 was initially assessed using multicolor immunohistochemistry (mIHC) in TLSs from 8 randomly selected tumor samples in the immunochemotherapy group (**Figure 3A**). We found that CD11c+ cells exhibited higher PD-L1 expression compared to CD68+ cells (**Figure 3B**, *p*=0.0078). Since CD11c+ primarily represents dendritic cells (DCs) or proinflammatory macrophages, this suggests that CD11c+ DCs are in close contact with T cells for antigen presentation (35). Next, we conducted a spatial analysis of CD11c+PD-L1+ cells and CD8+PD-1+ cells within TLSs (both peritumoral and intratumoral) using Halo software based on the mIHC results from 16 CR/MPR patient samples and 12 NR patients samples in the immunotherapy group (**Figure 3C**). The results suggested that the density of CD8+PD-1+ cells in TLSs decreased in association with treatment response status (**Supplementary Figure S2A**, *p*=0.0526). Additionally, the density of CD11c+PD-L1+ cells was significantly higher in the NR group compared to the pCR+MPR group (1327 ± 842.5/mm^2^ *vs*. 354 ± 4241.6/mm^2^, *p*<0.0001) (**Supplementary Figure S2B**). To investigate the proximity of PD-L1+ cells in the TME to TILs, we assessed the average distance between CD8+PD-1+ cells and CD11c+PD-L1+ cells. The average distance between these cells was shorter in NRs than in responders (**Figures 3D, E**, 22.93 ± 7.638 μm *vs*. 51.63 ± 35.45 μm, *p* = 0.0004). These findings suggest that PD1 inhibitor may block cell−cell interactions between the PD-L1−CD11c and PD1−CD8 axes in TLSs.

### Multipanel mass cytometry profiling reveals dynamic changes in immune cell populations before and after immunochemotherapy, with a focus on TLS-associated immune responses in resectable stage III NSCLC

**Figure 4:**
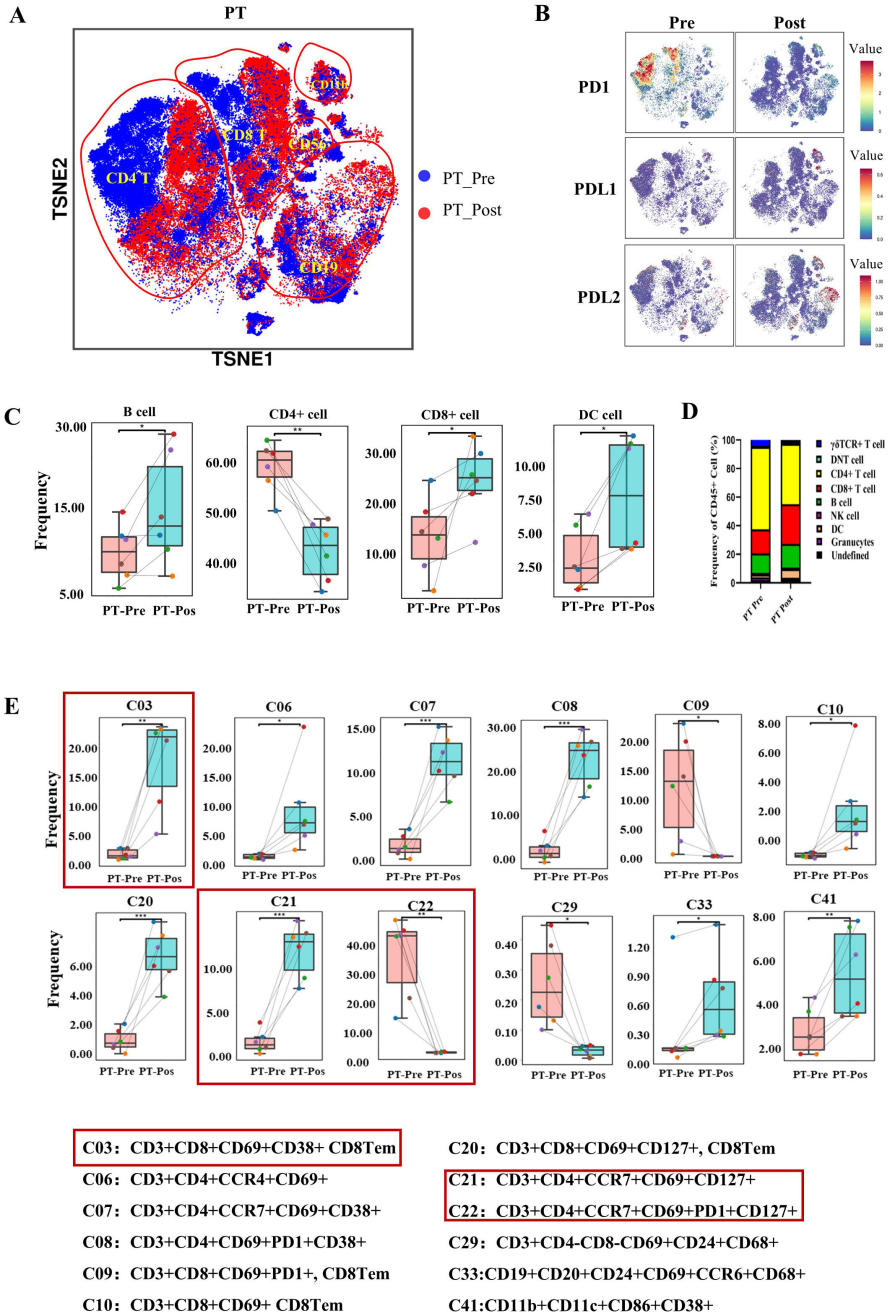
The immune landscape of patients with NSCLC before and after preoperative immunochemotherapy, as determined using CyTOF. **(A)** The t-distributed stochastic neighbour embedding (t-SNE) plot displays the overall distribution of different immune cell clusters between pre- and posttreatment in the primary tumor. **(B)** t-SNE plots showing PD-1, PD-L1 and PD-L2 expression on immune cells in primary tumors in the pre- and posttreatment groups. **(C)** Box plot comparing the relative abundance of 9 immune cell clusters pre- and posttreatment in primary tumors. **(D)** Composition of CD45+ immune cells (except for neutrophils) in primary tumors before and after treatment. **(E)** Bar plot showing the immune cell phenotype with a significant difference between pre- and posttreatment values in the primary tumor. The refined phenotypes identified from CyTOF analyses with significance are shown below the bar plots. The data are shown as the mean ± SD, n = 6. Paired t tests were performed to determine statistical significance. *p < 0.05, **p < 0.01, ***p < 0.001, ****p < 0.0001, ns, not significant.

To comprehensively assess overall immunological changes in resectable stage III NSCLC patients following treatment with immunochemotherapy, tumor-infiltrating lymphocytes (TILs) were profiled in 6 patients (**Table 1**). The blue color represents the baseline status, and the red color represents the posttreatment status. T-distributed stochastic neighbor embedding (t-TSNE) revealed that CD4+ T and CD8+ T cells compromised all PD-1+ T cells in PTs (**Figures 4A, B**). In contrast, the PD-L1 expression was observed on CD11c cells and CD11c-CD68+ cells, while PD-L2 was detected on CD19+ cells (**Figure 4B**).

Regarding the dynamic changes in major immune cell types following treatment, immune cells were clustered into 9 groups (granulocytes, DCs, NK cells, B cells, CD4+ T cells, CD8+ T cells, gdTCR+ T cells, double-negative T cells and undefined cells) (**Supplementary Figure S3A**). B cells, DCs and CD8+ T cells significantly increased after treatment in the PT group (**Figures 4C, D**). However, CD4+ T cells in the PT group significantly decreased after treatment (**Figures 4C, D**). Granulocytes, gdTCR+ T cells, DNT cells, NK cells and a group of undefined cells showed no significant changes in PTs after treatment. The comparison of the proportions of immune cell subtype proportions among CD45+ cells pre- and post-treatment in PTs is shown in **Figure 4D**.

The CyTOF panel, composed of 42 markers (**Table 2**), was able to identify more detailed immune cell subtype compositions, including 42 unique immune cell types (**Supplementary Figure S3B**) according to FlowSOM analysis. The number of three CD8+ T-cell clusters (C03: CD3+CD8+CD69+CD38+ CD8Tem, C10: CD3+CD8+CD69+ CD8Tem, and CD20: CD3+CD8+CD69+CD127+ CD8Tem) increased significantly, whereas one CD8+ T-cell cluster (C09: CD3+CD8+CD69+PD1+ CD8Tem) decreased significantly. Three CD4+ T-cell clusters (C06: CD3+CD4+CCR4+CD69+, C07: CD3+CD4+CCR7+CD69+CD38+, and C21: CD3+CD4+CCR7+CD69+CD127+) increased, and two CD4+ T-cell clusters (C08: CD3+CD4+CD69+PD1+CD38+ and C22: CD3+CD4+CCR7+CD69+PD1+CD127+) decreased. DCs (C41: HLA-DR+CD11b+CD11c+CD86+CD38+), B cells (C33: CD19+CD20+CD24+CD69+CCR6+CD68+), and gdT (C11: CD3+gdTCR+CD69+) increased, whereas DNTs (C29: CD3+CD69+CD24+CD68+) decreased (**Figure 4E**). Interestingly, no significant differences in B cells were observed in the PTs when considered as an individual cell group (**Figure 4E**). Other cell groups that were not significantly different are shown in **Supplementary Figure S4**.

### Mass cytometry reveals that CCR7+CD69+CD127+CD4+ cells are located close to TLSs, with decreased PD-1 expression potentially altering their function after immunochemotherapy

CyTOF revealed that the C21 cluster (CD3+CD4+HLA-DR+CD69+CCR7+CD127+) increased from 0% to 10% after immunochemotherapy treatment, while theC22 cluster (CD3+CD4+HLA-DR+CD69+CCR7+CD127+ PD1+) decreased from 40% to 0%. Upon comparing the markers for C21 and C22, we found that C21 may not represent a newly immerged cluster. Instead, it likely corresponds to the same cell group as C22, but with a decrease in PD1 expression.

**Figure 5:**
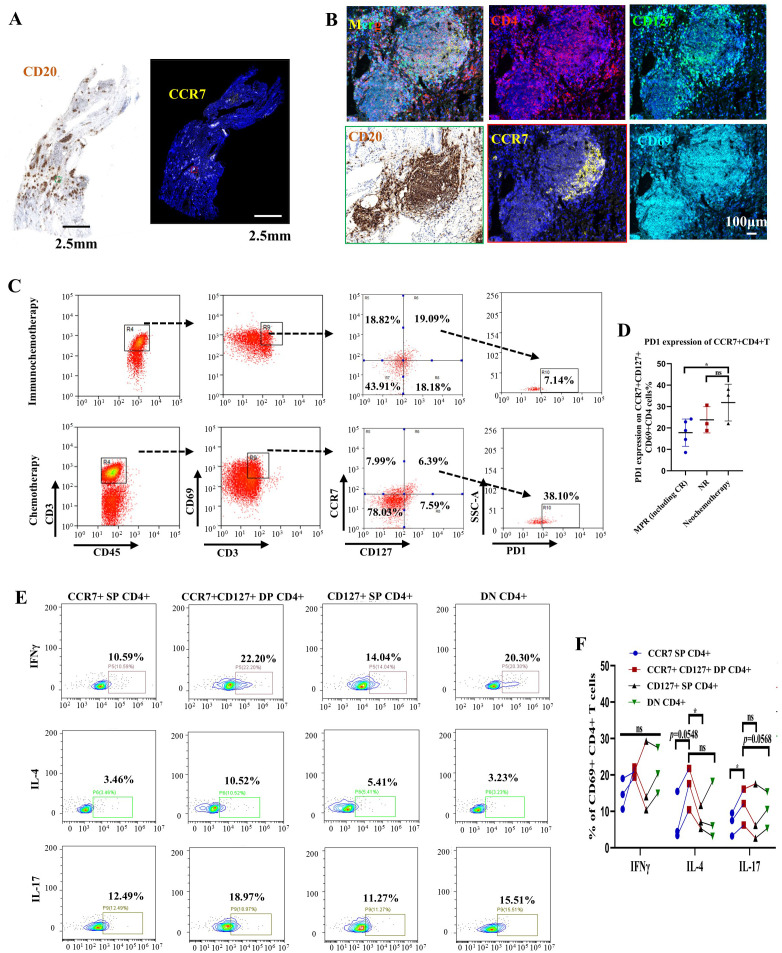
CCR7+CD127+CD4+ T cells near tertiary lymphoid structures (TLSs) might be involved in maintaining B-cell function in patients with NSCLC following immunochemotherapy. **(A)** Representative image of immunohistochemical (IHC) and immunofluorescence staining of serial tumor sections after treatment with a PD-1 inhibitor plus chemotherapy. Black scale bar=2.5 mm, white scale bar=2.5 mm. **(B)** Multiplex IHC staining of a representative tumor section showing the coexpression of CD4 (red), CD69 (cyan), CD127 (green), and CCR7 (yellow). White scale bar=100 μm. **(C)** Gating strategy for identifying PD-1 expression in CCR7+CD127+CD4 T cells subjected to immunochemotherapy (upper) or chemotherapy (lower). **(D)** Statistical analysis showing PD-1 expression on CCR7+CD127+CD4 subsets in primary tumors following immunochemotherapy (grouped according to pathological response) or chemotherapy. n≥3. **(E)** Representative flow cytometry plots showing IFNγ, IL-4, and IL-17 expression in different CD4+ T-cell subsets (CCR7+ SP CD4+, CCR7+CD127+ DP CD4+, CD127+ SP CD4+ and DN CD4+) isolated from primary tumors. **(F)** Statistical analysis showing IFNγ, IL-4, and IL-17 expression. n=3. Each dot represents an independent data point as determined by flow cytometry. The data are shown as the means ± SDs; n ≥3. Statistical significance was determined using one-way ANOVA with multiple comparisons in **(D, F)**. *p* < 0.05, **p < 0.01, ***p < 0.001, ns, not significant.

To evaluate the localization of this CD4+ cell subtype in tissue sections, we performed immunostaining and found that C21 is located close to TLSs (**Figures 5A, B**). Eight newly collected fresh tumor samples following immunochemotherapy (from 5 patients later assessed as MPR and 3 patients later assessed as NR, based on standardized pathology reports reviewed by multiple pathologists) and 3 samples following chemotherapy were used to evaluate PD-1 expression in CD3+CD4+CD69+CCR7+CD127+ cells. FACS sorting of this CD4+ T cells subtype showed that, consistent with CyTOF results, tumors that underwent chemotherapy exhibited higher PD-1 expression in C21 (which indicated that this group was C22), whereas tumors that received immunochemotherapy showed much lower PD-1 expression in C21 (**Figures 5C, D**, *p*=0.0369 and 0.2572, respectively). These findings suggest that the PD-1 checkpoint may block this CD4+ cell subgroup near TLSs.

To further evaluate whether this CD4+ cell subtype group has a regulatory effect on CD8+ T-cell, we assessed the secretion of Th1 (IFNγ), Th2 (IL-4), and Th17 (IL-17). The results confirmed that C21 cells exhibit greater IL-4 and IL-17 secretion than CD4+CD69+CD127+, CD4+CD69+CCR7+, or CD4+CD69+CD127-CCR7- cells (**Figures 5E, F**). These findings suggest that the C21 CD4+ T cell subgroup primarily assists B cells in functions related to Th2 and Th17 cells responses, compared to other subtypes of CD4+ T cells. The ability of these cells to secrete IL-4 also implies that they may contribute to maintaining TLS formation through B cells, although this hypothesis requires further investigation.

### Mass cytometry identifies CD38+CD8+ T cells induced by immunochemotherapy, which may compromise treatment efficacy

**Figure 6:**
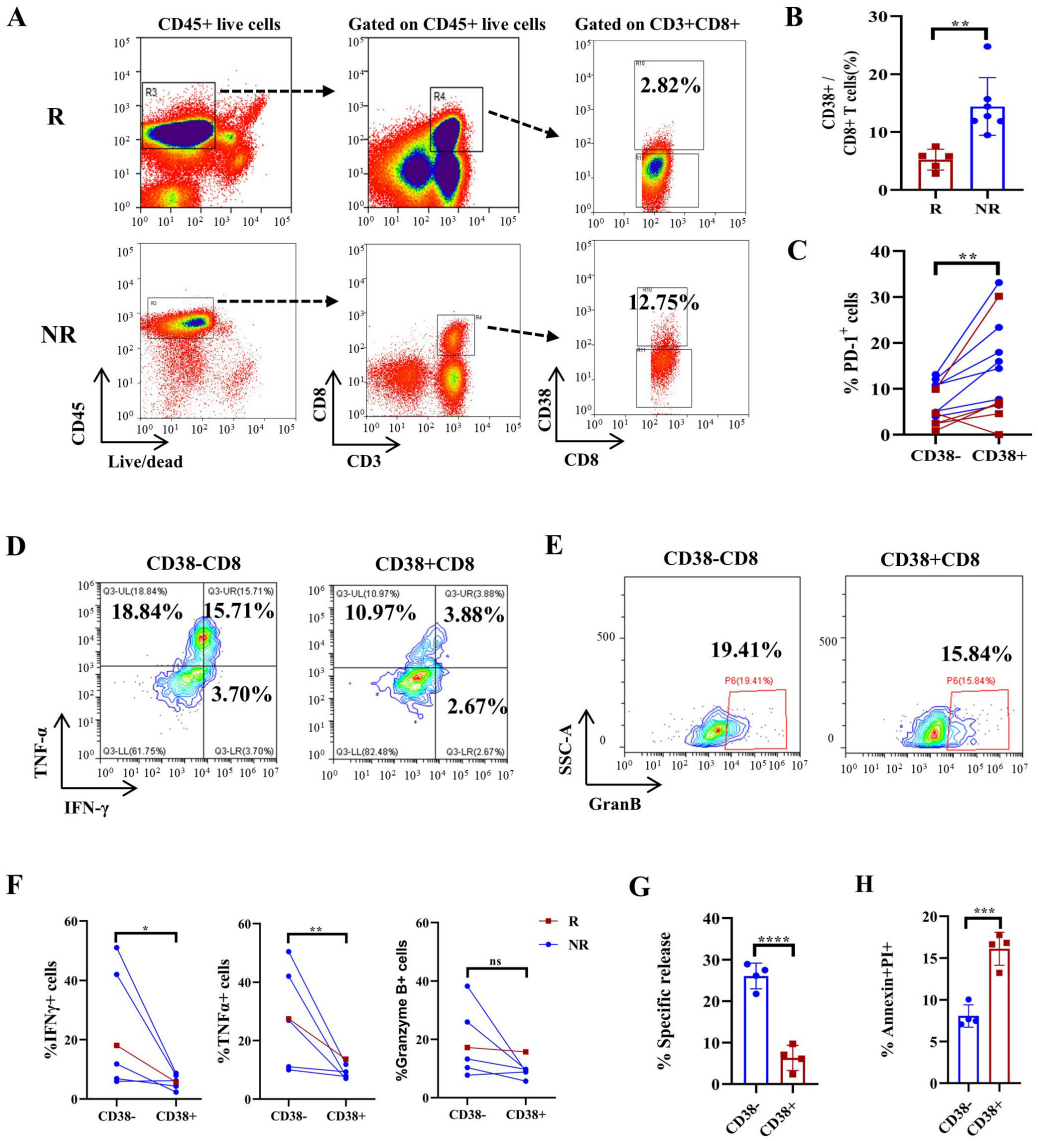
FACS-based quantification of CD38+CD8+ T cells predicts treatment response in an NSCLC cohort and CD38 overexpression-induced T-cell apoptosis and dysfunction. **(A)** Representative gating strategy for CD38+CD8+ T cells in responders (R) and nonresponders (NR) after immunochemotherapy. **(B)** Boxplots showing the proportion of CD38-positive CD8+ T cells in tumor tissue. n=12. **(C)** The proportion of PD-1-expressing CD38+ and CD38- T cells in tumor tissues. n=12. **(D)** Representative flow cytometry plots showing the expression of IFNγ and TNFα in CD38+ CD8+ and CD38- CD8+ T cells. **(E)** Representative flow cytometry plots showing the expression of granzyme B in CD38+ CD8+ and CD38- CD8+ T cells. **(F)** Statistical analysis showing IFNγ, TNFα and granzyme B expression in responsive patients and nonresponsive patients. n=6. **(G)** Specific analyses using EuTDA cytotoxicity assays. **(H)** Boxplots showing the proportions of PI/Annexin-V+ cells among control CAR-T cells and CD38 OE CAR-T cells in **(G)** The data are shown as the means ± SDs, n = 6. Mann−Whitney tests were performed to determine statistical significance. Paired t tests were performed to determine statistical significance in **(C, F)** *p < 0.05, **p < 0.01, ***p < 0.001, ****p < 0.0001, ns, not significant.

Our CyTOF data revealed a significant increase in the frequency of CD38+CD8+ (C03) T cells in PT sections after immunochemotherapy treatment (**Figure 4E**). Specifically, the percentage of CD38+CD8+ T cells significantly increased in NR patients following immunochemotherapy (**Supplementary Figure S5**). To assess the clinical significance of CD38+CD8+ T cells, we sorted CD38+CD8+ cells from newly collected fresh PT samples from 5 patients later assessed as pCR+MPR and 7 patients later assessed as NR, based on standardized pathology reports reviewed by multiple pathologists. We found that PTs from NR patients had substantially more CD38+CD8+ T cells than those from pCR+MPR patients (**Figures 6A, B**, *p*=0.0029). Moreover, CD38+CD8+ T cells exhibited much higher PD-1 expression compared to CD38-CD8+ T cells in PTs (**Figure 6C**, *p*=0.0063). Functional analyses revealed that CD38+CD8+ T cells produced lower levels of IFNγ and TNFαthan CD38-CD8+ T cells (**Figures 6D, F**, *p*=0.0145 and *p*=0.0033, respectively), though no significant difference in granzyme B secretion was observed (**Figures 6E, F**, *p*=0.0702). To explore how CD38 expression influences T-cell function, we generated CD133-specific CAR-T cells overexpressing CD38 based on our previous study (33) and compared the cytotoxicity. At an effector/target (E:T) ratio of 1:1, CD38-expressing CAR-T cells showed reduced cytotoxicity than CD133-specific CAR-T cells against the CD133+ A549 cell line (**Figure 6G**, *p*<0.0001). One potential reason is that CD38 expression induced CD8+ T-cell apoptosis (**Figure 6H**, *p*=0.0005).

**Figure 7:**
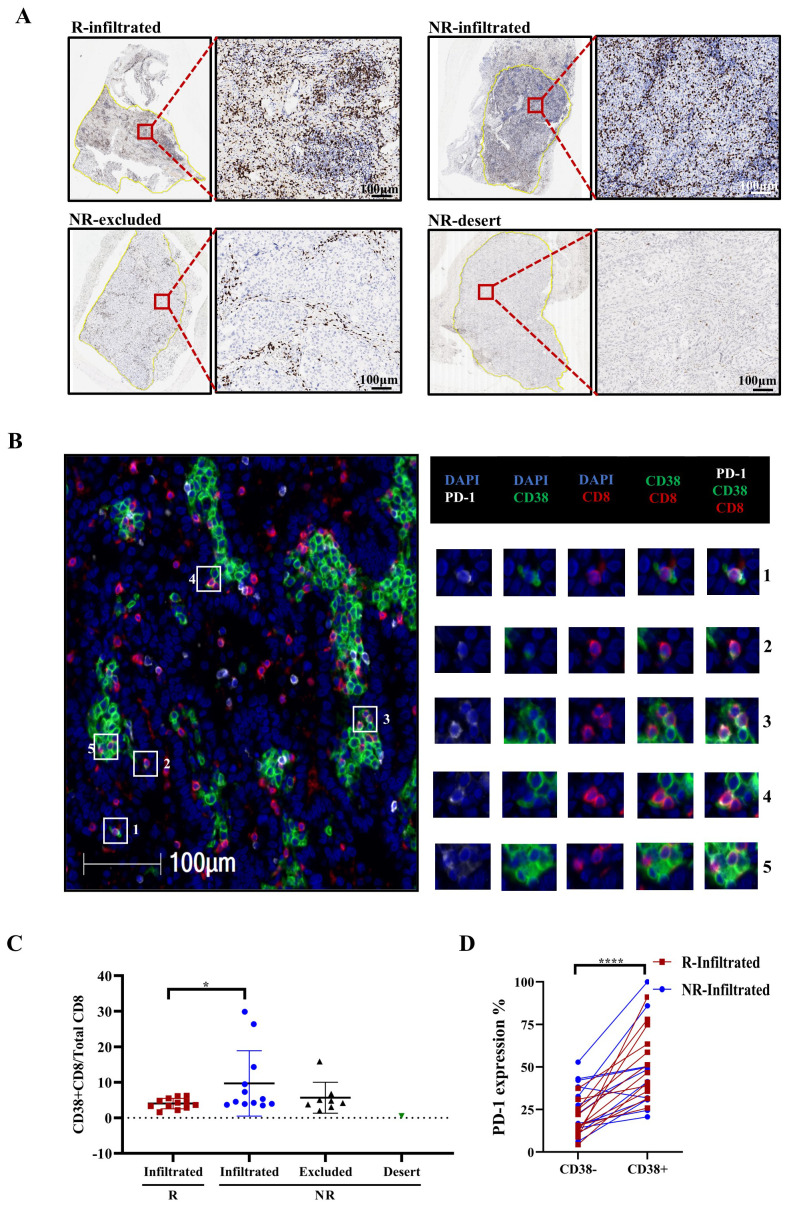
Multiplex immunohistochemistry (mIHC) revealed that the proportion of CD38+ CD8+/CD8+ T cells corresponded with no response. **(A)** T-cell infiltration defined by immunohistochemistry (IHC) staining. Representative IHC images of CD8+ T cells in different patient groups, classified according to CD8+ T-cell density and distribution. The black and white scale bars represent 100 μm, respectively. **(B)** mIHC staining of a representative tumor section showing the co-expression of PD-1 (white), CD38 (green), and CD8 (red). Squares 1-5 show representative CD38+CD8+ T cells with PD-1 *colocalization*. White scale bars=100 μm. **(C)** Frequency of CD38 expression on CD8+ T cells in different patient groups. R=responder, NR=non-responder. Mann−Whitney tests were performed to determine statistical significance. n=12. ns = not significant, *p < 0.05, **p < 0.01, ***p < 0.001, ****p < 0.0001; ns, not significant. **(D)** Frequency of PD-1 expression on CD38- and CD38+ CD8+ T cells. n=24 (n=12 for both R and NR-infiltrated groups). ns = not significant, *p < 0.05, **p < 0.01, ***p < 0.001, ****p < 0.0001; ns, not significant.

Next, we performed CD8 staining in tumor sections from patients who underwent immunochemotherapy to classify them into four groups based on CD8+ T-cell infiltration: response with CD8+ T-cell infiltration (R-infiltrated), no response with CD8+ T-cell infiltration (NR-infiltrated), no response with CD8+ T-cell infiltration surrounding the tumor (NR-excluded), and no response with minimal CD8+ T-cell staining (NR-desert) according to a previous study (36) (**Figure 7A**). We then performed mIHC staining in these tumor sections to analyze the co-localization of PD-1, CD38 and CD8, and further compare the expression of CD38 and PD-1 on CD8+ cells across different patient groups (**Figure 7B**).

Our mIHC data revealed that NR-infiltrated patients had a higher ratio of CD38+CD8+ cells to total CD8+ cells than R-infiltrated patients (**Figure 7C**, *p*=0.0465). Furthermore, PD-1 expression was much higher on CD38+CD8+ cells in the immunochemotherapy group, as shown in **Figure 7D** (p<0.0001). Collectively, these data suggest that CD38+CD8+ T cells may reduce their antitumor effect after neoadjuvant immunochemotherapy.

## Discussion

The tumor microenvironment (TME) plays a critical role in antitumor processes. This study investigated the emergence and function of tertiary lymphoid structures (TLSs) and key immune cell subsets within the TME using IHC/mIHC, flow cytometry, and CyTOF. Our data highlight the significance of TLSs and these immune cell subgroups in the response to immunochemotherapy in patients with locally advanced NSCLC. Both chemotherapy and immunochemotherapy induced similar numbers and areas of TLS in NSCLC patients. However, a higher rate of pCR and MPR was observed when PD-1 inhibitors are added (5). In addition to reinvigorating PD-1+ T cells (37), we hypothesize that PD-1 inhibitors may enhance the antitumor effect of TLSs. Consistent with findings by Goc, J. et al., who demonstrated that mature dendritic cells (DCs) within TLSs elicit an efficient antitumor CD8+ T-cell response (38), our spatial distance analysis revealed that patients achieving pCR+MPR had fewer interactions between CD11c+PDL1+ DCs and CD8+PD1+ T cells compared to non-MPR patients following neoadjuvant immunochemotherapy. These results suggest that a PD-1 inhibitor may disrupt the interaction between CD11c+PDL1+ DCs and CD8+PD1+ T cells, thereby enhancing CD8+ T-cell function within TLSs. This aligns with previous studies indicating that PD-L1 expression on DCs can negatively affect CD8+ T-cell responses (34, 39) and underscores the importance of immune checkpoints in myeloid cells as key regulators of immune checkpoint inhibitor efficacy.

Previous studies have shown that the prognostic effect of CD8+ TILs is significantly enhanced in the presence of B cells (40). Since CD20+ B cells are key components of TLSs, the cooperative interactions between these lymphocyte subsets may contribute to more potent antitumor immunity (41). In line with this, our results showed that cytotoxic CD8+ T cells are enriched near the TLS areas. Using CyTOF, we identified a CCR7+CD4+ T-cell subset in proximity to TLSs, and functional analyses indicated that PD-1 inhibitors may help CCR7+ CD4+ T cells support Th2 and Th17 functions, potentially restoring TLS function through B-cell involvement. A recent study reported that a population of stem-like CD4+ T cells residing in TLSs can replenish effector cells independent of secondary lymphoid organs, particularly in vasculitic arteries. These stem-like CD4+ T cells give rise to two effector populations: eomesodermin (EOMES)+ cytotoxic T cells and B-cell lymphoma 6 (BCL6)+ T follicular helper-like cells (42). This finding is intriguing, as it suggests that TLSs in the tumor microenvironment may harbor similar stem-like CD4+ T cells to those observed in autoimmune vasculitis, potentially contributing to the local immune response and tumor control.

The emergence of dysfunctional CD38+CD8+ T cells following neoadjuvant immunochemotherapy, as observed in this study, may compromise treatment effectiveness. Previous studies have suggested that the optimal priming of CD8+ T cells is essential for the success of anti-PD-1 monotherapy. For example, when PD-1 blockade is administered first and followed by a vaccine, dysfunctional CD38+CD8+ cells can emerge, potentially impairing treatment effectiveness (43–45). However, our study found that even when chemotherapy is combined with PD-1 blockade, CD38+CD8+ T cell was observed in patients who did not achieve MPR, suggesting the presence of suboptimally primed CD8+ T cells. These discrepancies highlight the need for further investigation into the effects of combining anti-PD-1 therapy and chemotherapy. Exploring the sequencing of treatments for future research, such as administering neoadjuvant chemotherapy first, followed by sequential PD-1 blockade. Neoadjuvant chemotherapy alone has also been shown to induce TLS formation, which may influence subsequent immune responses (46). The PD1+CD38+ phenotype, without adequate antigenic stimulation, leads to a terminally dysfunctional state with a lack of effector functions and memory generation, resulting in T-cell apoptosis upon rechallenge. Moreover, CD38, a key NAD+ glycohydrolase (NADase), affects T-cell activation and differentiation by disrupting signaling and metabolic processes (47–49). The impact of the CD38–NAD+ axis on chromatin remodeling and T-cell response reprogramming warrants further investigation. Therefore, the combination of chemotherapy and PD-1 blockade plays a pivotal role in determining therapeutic outcomes. The PD-1+CD38+ phenotype of CD8+ T cells could serve as both a predictive biomarker and therapeutic target for enhancing the efficacy of chemotherapy and anti-PD-1 treatments.

Overall, B cells increased after treatment in the PT group. However, no significant changes were observed when the data were analyzed in more refined subgroups. The observed heterogeneity in B cell responses and their potential antitumor functions have been reported in other studies (50, 51). This lack of significant findings may be partially attributed to the limitations of the CyTOF approach used in this study, which analyzed only 42 markers. Future studies should incorporate more advanced techniques, such as single-cell sequencing and spatial transcriptomics. These methods will allow for a more comprehensive understanding of how B cells collaborate with T cells in the tumor microenvironment during immunochemotherapy treatment.

One limitation of this study was that we only compared immunochemotherapy with chemotherapy alone, without evaluating anti-PD-1 monotherapy independently. Anti-PD-1 monotherapy was not independently evaluated due to its low pathologic response rate compared to combined treatment (52). Further investigation is needed to determine whether PD-1 inhibitors alone can induce the emergence of TLSs, as observed with chemotherapy or immunochemotherapy. Previous studies have shown that neoadjuvant PD-1 blockade induced TLS emergence in most patients who achieved MPR or cPR, while TLSs were not detected in NR patients (29). Given that TLSs have prognostic effect in antitumor responses, future research should focus on strategies to promote TLS formation within tumors, potentially through TLS-associated cytokines and chemokines such a as lymphotoxin (53), TNFα (54) and CXCL13 (55), in combination with PD-1 checkpoint blockade. Another limitation of this study was that the lack of assessment of the CD4+CCR4+ phenotype, which has been shown in several studies to negatively affect PD-1 checkpoint blockade responses (56–58) was not assessed in our study.

Despite these limitations, our study demonstrated that chemotherapy can induce TLS formation and that the addition of PD-1 blockade enhances TLS function. Specifically, CD4+CCR7+ cells and a longer distance between the PD-L1–DC axis and PD-1+CD8+ T cells within TLSs may have positive impact on treatment outcomes, while CD38+CD8+ T cells appear to serve as a negative marker of therapeutic response. Furthermore, since all samples in our study were from clinical node-positive patients, this work provides a comprehensive understanding of the TME during neoadjuvant immunochemotherapy, highlighting key immune cell dynamics and their potential influence on therapy.

## Conclusions

Neoadjuvant immunochemotherapy has significantly reshaped the treatment approach for resectable NSCLC. Our study highlights TLSs and immune cell markers as potential predictors of treatment response. Notably, the PD-1-PD-L1 distance within TLSs during immunochemotherapy offers potential clinical applications. The altered function of PD-1+ on CD4+ cells near TLSs, coupled with reduced PD-1 expression, may further influence treatment outcomes. Additionally, the emergence of CD38+ CD8+ cells correlates with poor treatment response. These findings underscore the potential to tailor neoadjuvant immunochemotherapy, optimizing immunotherapeutic strategies to enhance efficacy in resectable NSCLC.

## Data Availability

The original contributions presented in the study are included in the article/**Supplementary Material**. Further inquiries can be directed to the corresponding authors.
