# Supplementary material for: TLS and immune cell profiling: immunomodulatory effects of immunochemotherapy on tumor microenvironment in resectable stage III NSCLC
Source: Front Immunol. 2024 Dec 11;15:1499731. doi: 10.3389/fimmu.2024.1499731 (PMC11670196; doi:10.3389/fimmu.2024.1499731)
Supplement: Supplementary file 1 [file DataSheet1.docx]

**TLS and immune cell profiling: Immunomodulatory effects of immunochemotherapy on tumor microenvironment in resectable stage III NSCLC**

Chaopin Yang, Jinqi You, Yizhi Wang, Si Chen, Yan Tang, Hao Chen, Haoran Zhong, Ruyue Song, Hao Long, Tong Xiang, Ze-Rui Zhao and Jianchuan Xia

Supplementary figures and figure legends2

**Supplementary figures and figure legends**


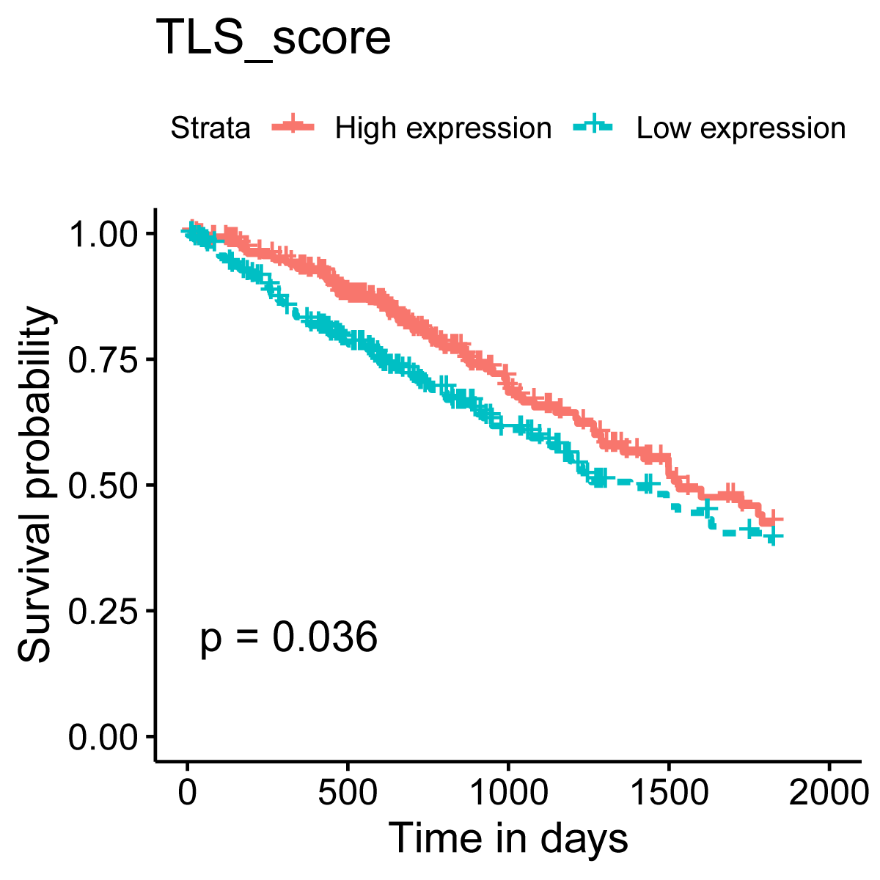


**Figure S1.** Kaplan‒Meier survival curves for 526 NSCLC patients with lung adenocarcinoma in public databases stratified according to TLS score. Patients were divided into two groups by the median TLS score, which was calculated by the expression of 26 genes. The p value was estimated based on the log-rank test.

**
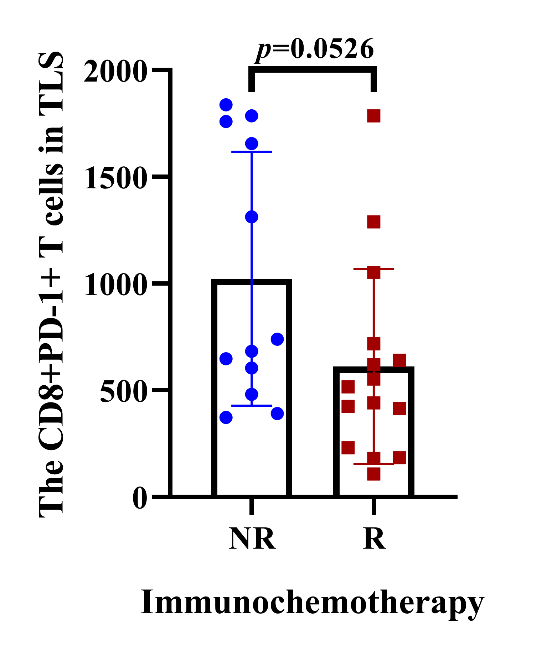

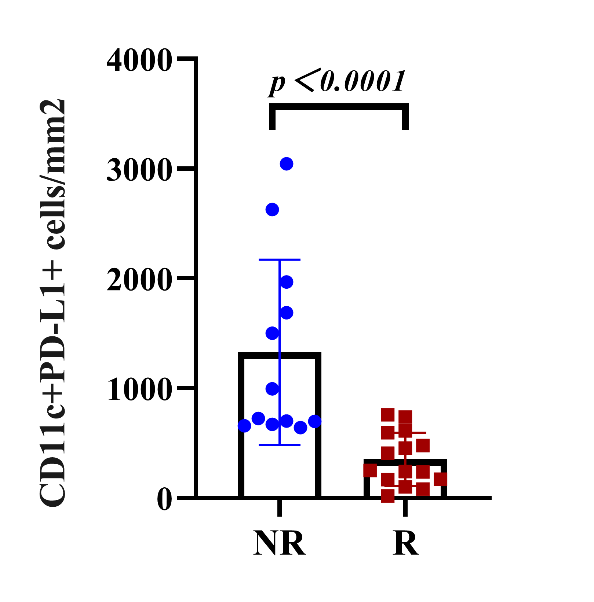
a b**

**Figure S2.** Bar plot showing quantification of CD8+PD-1+ (a) and CD11c+PD-L1+ cells (b) per square millimeter in TLSs. Mann‒Whitney tests were performed to determine statistical significance.


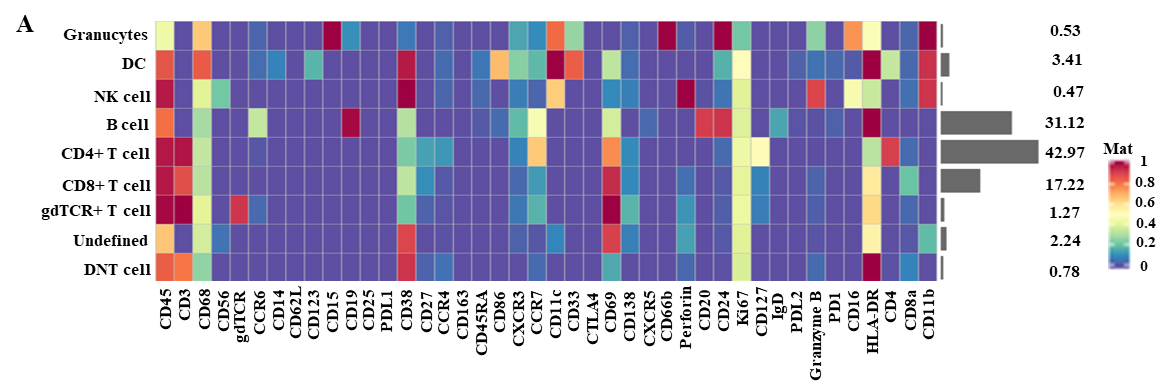
**a**

**
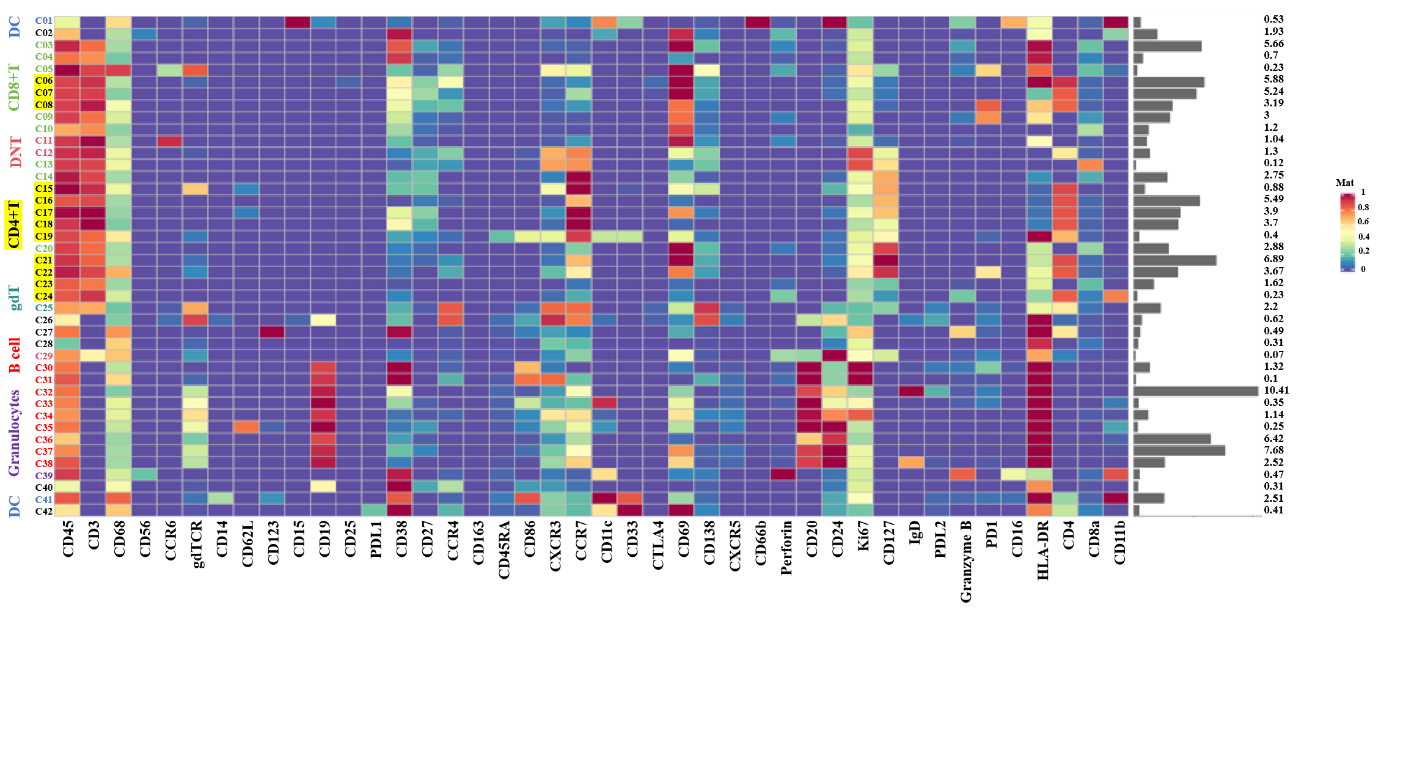
b**

**Figure S3.** Heatmap showing the normalized marker expression for the 9 immune cell populations (a) and 42 identified cell populations (b) grouped by manual merging of the 42 metaclusters generated by FlowSOM.

**
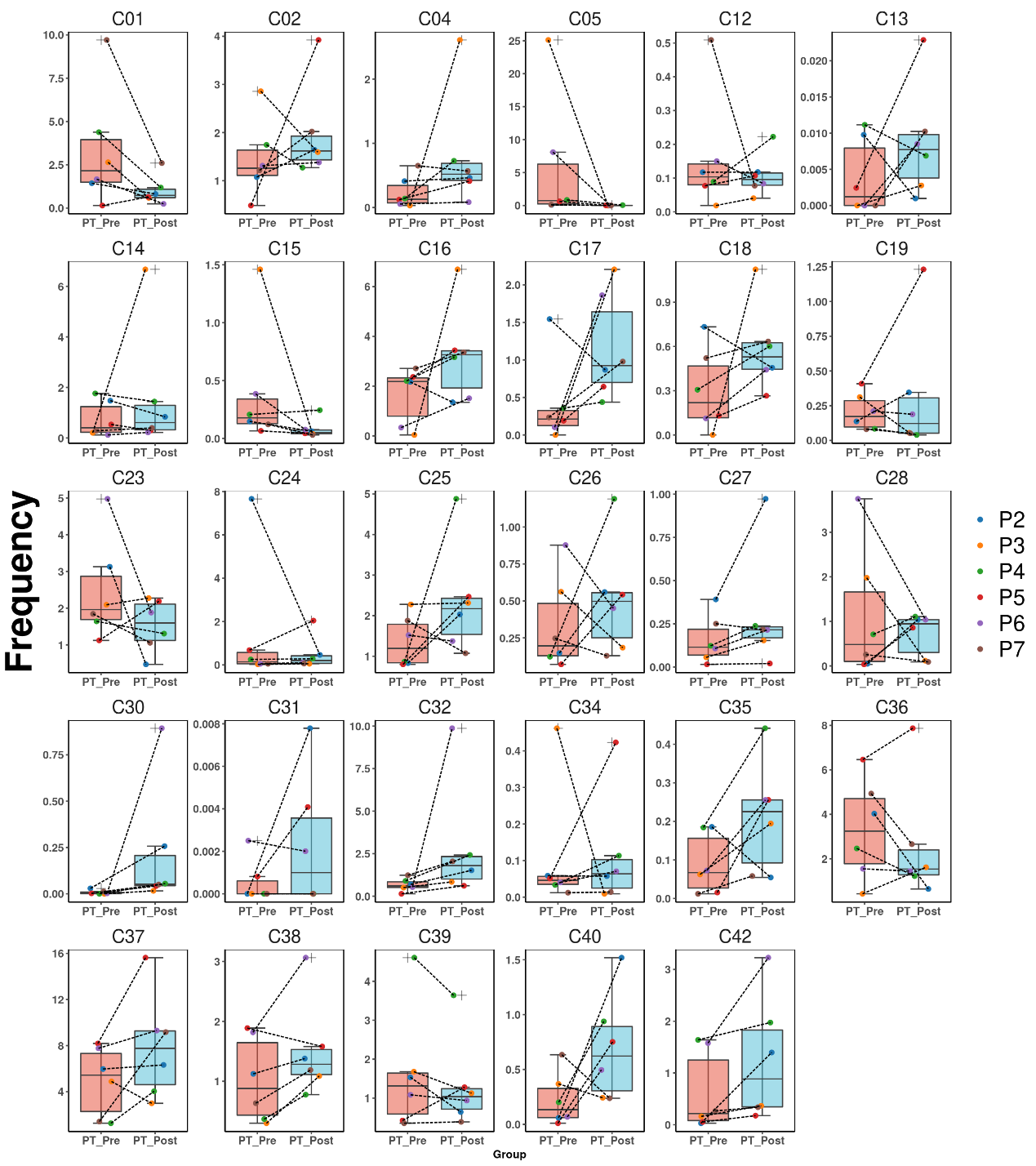
**

**Figure S4.** Box plot showing the frequencies of immune cell subtypes illustrated by CyTOF comparing pretreatment and posttreatment in primary tumors without significant differences. Paired t tests were performed to determine statistical significance.


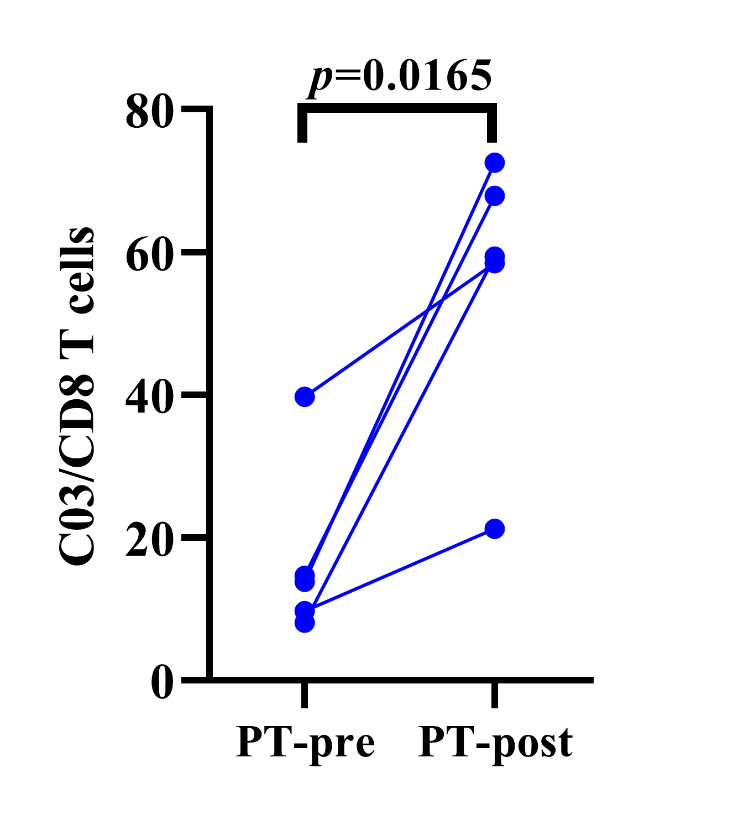


**Figure S5.** Bar plot showing the C03 immune cell type with a significant difference between pre- and posttreatment in the primary tumor of nonresponders (NR).
